# Prognostic biological factors in severe acute pancreatitis

**Published:** 2014

**Authors:** CC Popa

**Affiliations:** * „Carol Davila” University of Medicine and Pharmacy, Department of Surgery, 2nd Surgery Clinic, University Emergency Hospital Bucharest, Romania

**Keywords:** acute pancreatitis, severity, biological, prognostic

## Abstract

Acute pancreatitis is a serious disease. Many clinical and laboratory prognostic scores for the severity of acute pancreatitis have been proposed over the years. The aim was to identify the biological factors of prognostic severity. The study was prospective, including a four-year period between 2007 and 2010. 103 patients were diagnosed with severe acute pancreatitis and treated in a surgical clinic in Bucharest. 58 were males, accounting for 56.31%, and 45 were women, 43.69% respectively. Numerous biochemical analyses of blood, especially the number of leukocytes, glucose, urea and bilirubin were monitored. They proposed generic profiles for patients with severe acute pancreatitis. Conclusions: There is no single biological prognostic factor, but a combination of different markers may contribute to a more precise prediction of severity, as confirmed by international literature.

TAcute pancreatitis is an acute inflammatory process of the pancreas gland acinar cells. The main objective of estimating the severity of acute pancreatitis is to distinguish between the mild or moderate forms of development of the severe forms, which have a serious evolution potential and are accompanied by a high mortality rate. Numerous prognostic factors in severe acute pancreatitis have been identified in the last decades of time. Our study was prospective, including a four-year period between 2007 and 2010. The aim of this study was to present the experience of the general surgery clinic in an emergency hospital in Bucharest regarding the identification of prognostic biological factors in severe acute pancreatitis.

TA positive diagnostic and assessment of severity of acute pancreatitis was based on etiological, clinical, laboratory (blood and urine biological analysis), imaging, pathological and intraoperative criteria. The present study followed blood biochemical data such as CBC, ESR, fibrinogen, glucose, urea, creatinine, transaminases (AST, ALT), bilirubin (total, direct), ionogram (Na, K, Cl), calcium, LDH, blood gases (pH, pO2, pCO2), total cholesterol.

TThe statistical analysis was performed by including variables sought to highlight the impact of biological risk factors on the development of mild or severe acute pancreatitis. SPSS 10.0 statistical software for Windows was used for the coordination of data and statistical tests, with software like Word, Excel and Epi Info. Data were expressed as numbers, averages, standard deviations, ranges and percentages.

TOf the 238 patients diagnosed with acute pancreatitis and its complications and treated in the clinic in 2007-2010, 103 patients (43.28%) presented severe acute pancreatitis and 135 patients (56.72%) mild acute pancreatitis (**Fig. 1**).

**Fig. 1:**
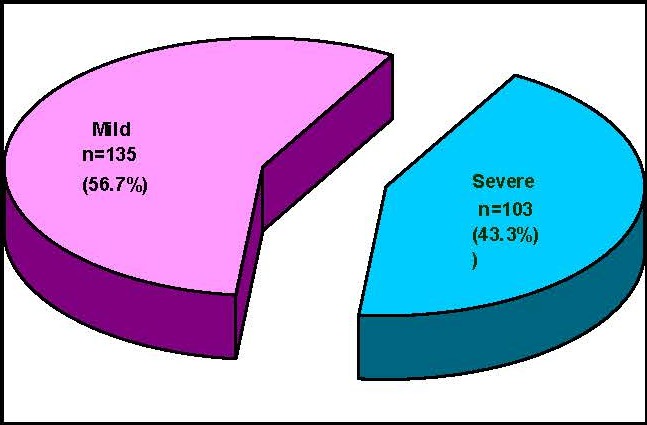
Distribution of patients according to severity

As far as the distribution according to gender is concerned, of the 103 patients with severe acute pancreatitis, 58 were men and 45 women, representing 56.31% and 43.69% that have been read, so the disease can affect both sexes equally, with some extra chance to affect men more than women (**Fig. 2**).

**Fig. 2:**
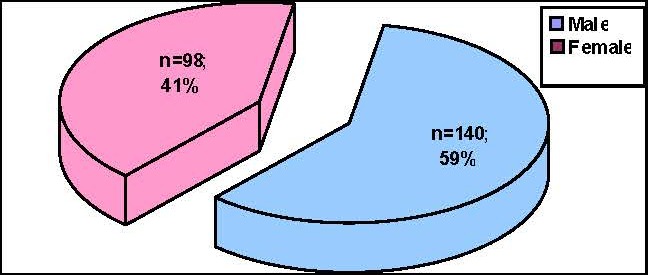
Distribution of patients according to gender

The average age of patients in the study group was 52.43 years and had limits between 23 years and 84 years. As far as the distribution according to gender is concerned, the average age of women was 55.36 years, with a range between 23 years and 82 years and the average age for men was 50.38 years, with a range from 25 years to 84 years. Depending on the severity of acute pancreatitis, the median age in patients with mild form was 50.5 years and in patients with severe form of 55.0 years. For severe forms, the number of male patients was higher in the range of 36-65 years and of the female between 36-45 and 66-75 years (**Fig. 3**).

**Fig. 3:**
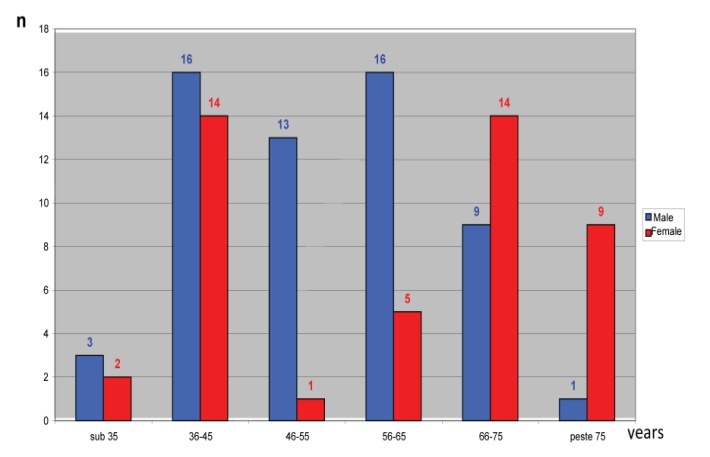
Distribution of patients with severe acute pancreatitis according to gender and decade of age

For a number of parameters averages and standard errors for each of the two clinical forms of pancreatitis, mild or severe, were calculated. Inter-group comparison (mild / severe) was made by using the t test and the resulting differences (mean difference) are statistically significant (p <0.001) in favor of the servers (**Table 1**).

**Table 1:** Distribution averages and standard errors for several biological parameters and age, depending on disease severity

	**MILD**		**SEVERE**	
**Variable**	**Media**	**Error Std.**	**Media**	**Error Std.**
Age (years)	50.5	1.3	55.0	1.4
Leukocytes ( /mm3)	6787.4	137.5	13722.3	328.1
Hemoglobin (g/dl)	14.2	0.2	10.1	0.2
Glucose (g%)	97.7	1.4	171.9	6.1
Urea (mg%)	35.7	0.6	94.5	7.1
Creatinin (mg%)	0.9	0.1	2.2	0.1
Na (mEq/l)	142.9	0.7	133.0	0.6
K (mEq/l)	4.2	0.1	3.2	0.1
Cl (mEq/l)	100.2	0.4	96.1	0.7
GOT (UI)	36.3	2.3	149.7	8.4
GPT (UI)	42.2	2.6	164.5	8.9
Total bilirubin (mg%)	1.1	0.1	2.0	0.1
Direct bilirubin (mg%)	0.4	0.1	1.2	0.1
pH	7.39	0.01	7.30	0.01
pO2 (%)	95.2	0.5	66.2	1.2
pCO2 (%)	37.6	0.5	34.7	0.7
LDH (mg%)	167.4	2.7	307.3	6.9
ESR (mm/1h)	12.4	0.7	44.1	1.8
Fibrinogen (mg%)	343.5	7.4	614.5	11.1
Calcium (g%)	8.8	0.1	7.5	0.1
Cholesterol (mg%)	200.9	2.2	242.8	3.5

Based on the results obtained by calculating the averages and standard errors for multiple parameters for each of the two clinical forms of pancreatitis, mild or severe, generic profiles of patients could be created as it follows:

- patient with mild pancreatitis was about 50 years old and presented WBC of 7,000/mm3 on admission, hemoglobin of 14.2g/dl, glucose 98 mg%, urea 36 mg%, total bilirubin 1.1 mg%, pH 7.39, pO2 95%, cholesterol 201 mg%, and liver enzymes and ionogram were normal;

- patient diagnosed with severe pancreatitis was about 55 years, WBC of 13,700/mm3, hemoglobin of 10.1g / dL, glucose 172 mg%, urea 95 mg%, total bilirubin 2 mg%, pH 7.30, pO2 66%, cholesterol 243 mg%, changes in liver enzymes, electrolyte imbalances.

Patients with severe acute pancreatitis have a WBC which is significantly higher from the statistical point of view than that of patients with mild acute pancreatitis; they also have elevated blood glucose, urea, creatinine, GOT, GPT, total bilirubin, direct bilirubin, LDH, ESR, fibrinogen and cholesterol, all these values being of p <0.001.

On the other hand, patients with severe acute pancreatitis have significant lower values from the statistical point of view than patients with mild acute pancreatitis in the case of hemoglobin, sodium, potassium, chloride, pH, pO2's, pCO2’s and calcium, for all the values of p <0.001.

Changes in their biological constants in the development of acute pancreatitis were detailed later.

30 patients (29.13%) with severe acute pancreatitis had values of over 16,000 leukocytes/mm3, but no patient with mild acute pancreatitis registered these elevated leukocytes (**Fig. 4**).

**Fig. 4:**
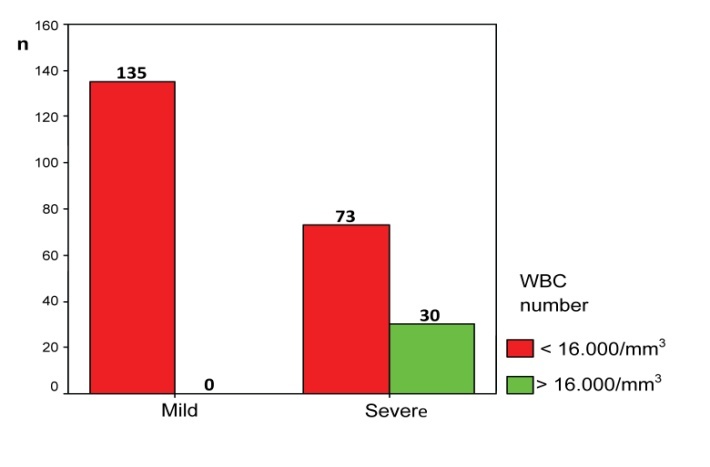
Distribution of patients according to the disease severity and the number of leukocytes

1 mg% bilirubin values prevailing in severe pancreatitis (90 patients, 90/103, 87.38%) is uncommon in mild cases (46 patients, 46/135, 34.07%) as shown in **Fig. 5**.

**Fig. 5:**
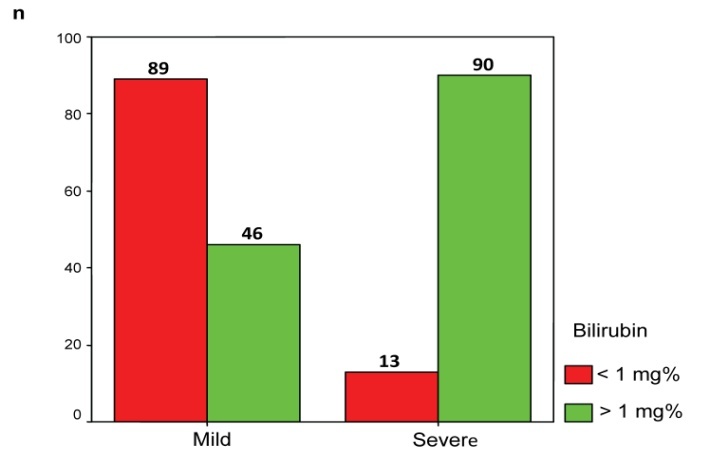
Distribution of patients according to the diesase severity and bilirubin values

The graph below (**Fig. 6**) shows the development of blood glucose values above 200 mg%. 35 patients with severe form (35/103, 33.98%) showed an increase in glucose over those opposed to any patient with mild form.

**Fig. 6:**
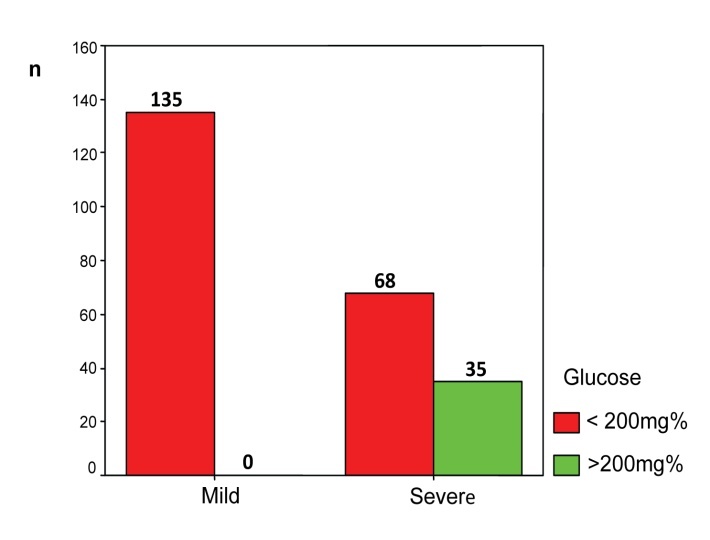
Distribution of patients according to the disease severity and blood glucose

Urea values increased above 100 mg% only in severe acute pancreatitis, respectively 27 patients (27/103, 26.21%); in mild form, all urea values being below 100 mg% (**Fig. 7**). Our study showed the presence of acute renal failure in 84 patients with severe acute pancreatitis (84/103, 81.55%), compared to only 10 cases (10/135, 7.41%) in patients with mild acute pancreatitis. Unlike acute renal failure, the chronic disease has been found to be associated in 8 patients with severe form of acute pancreatitis (8/103, 7.77%) and only 2 patients with mild form (2/135, 1.48%).

**Fig. 7:**
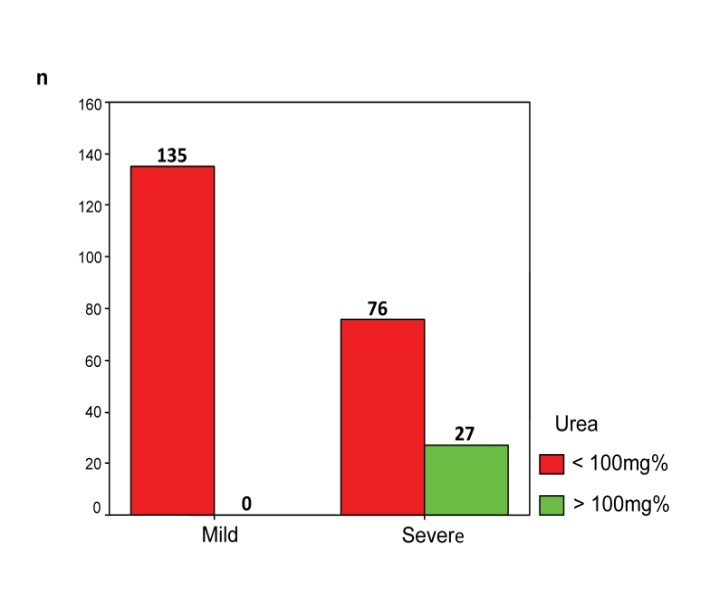
Distribution of patients according to the disease severity and the urea level

## Discussion. Conclusion

• The identification of prognostic biological factors in severe acute pancreatitis is a concern which appeared earlier in Romanian literature [**1**].

• In the group studied, the percentage of patients with severe acute pancreatitis of all those with acute pancreatitis is similar to the international literature [**2**].

• The average age of patients in the study group is similar to those in international studies [**2**,**3**]. In most studies, the advanced age is a negative prognostic factor in acute pancreatitis [**3**].

• Leukocytosis is a predictive marker of severity, especially if discovered in the first and seventh days after admission [**4**]. Analyzing the Romanian literature, Costea and Neagu published a study in 2012 about the importance of multifactorial leukocytosis in monitoring patients with severe pancreatitis in ICU [**5**].

• Bilirubin was introduced in the context of clinical and biochemical severity scores due to the recognition of the increased bilirubin in organ dysfunction generally and in the liver dysfunction especially. In these scores, bilirubin values above 1 mg% is considered abnormal, and values above 3 or 4 mg% indicates severe hepatobiliary damage [**6**].

• It is known in the international literature that glucose is increased at admission and is an early prognostic factor of severity of acute pancreatitis [**7**]. Being a highly sensitive parameter of severity, it has been included in various clinical and biochemical scores. Of these, Hong Kong score is based only on glucose and urea, but has a degree of prediction of severity like other scores, being more complicated [**8**].

• Increased urea appears as renal failure of severe acute pancreatitis default multiple organ failure. Numerous clinical and biochemical severity scores analyze elevated urea and monitor them in various stages of the disease. Hong Kong score based only on urea and glucose shows the important role in the classification of severity of acute pancreatitis [**8**]. Patients with increased urea at admission require more specialized treatment days in ICU. In addition, mortality in patients with increased urea was found significantly elevated [**9**].

• In conclusion, our analysis confirms that there is no single biological prognostic factor and only a combination of different markers may contribute to a more precise prediction of severity, as confirmed by Cochior and Constantinoiu, analyzing the Romanian literature [**10**] or by different authors in international literature [**11**,**12**].
